# State Medical and Chirurgical Society

**Published:** 1855-09

**Authors:** J. Howes

**Affiliations:** Rec. Sec’y.


**MEETING OF THE STATE MEDICAL AND CHIRURGICAL SOCIETY AT MUSCATINE, JUNE 14, 1854.**

Society met. Dr. Reader in the Chair. The Secretary being absent, Dr. G. R. Henry was appointed Secretary pro tem.

Moved that the President fill by appointment, the board of Censors. The following gentlemen were then appointed, viz: Drs. M'Gugin, Arnold, and Wainwright. The board retired.

The board, upon return recommended that the following gentlemen be elected members of the Society.

Dr. Freeman Knowles, Keokuk ; Dr. J. M. Robertson, Louisa Co. ; Dr. John Cleves, do ; Dr. William Clark, do ; Dr. H. T. Cleaver, do ; Dr. C. L. Chambers, Tipton ; Dr. H. C. Sanford, Montezuma ; Dr. Noah Green, Rochester Co. ; Dr. J. Howes, Burlington ; Dr. B. P. Rankin, Keokuk ; Dr. John O. Skinner,

Charleston; Dr. J. H. Hershy, Muscatine; M. W. Wilson, Saratoga, N. Y.; E. J. Fountain, N. Y.; Dr. B. W. Thompson; Dr. Simon Schock, Muscatine; Dr. James M. Hamilton, do.

On motion, the report was received and adopted.

The committee on nominations, nominated the following persons as officers for the ensuing year:

President, Dr. Geo. Reeder; 1st Vice President, Dr. J. M. Robertson; 2d Vice President, Dr. M'Gugin; Corresponding Secretary, Dr. C. L. Chambers; Rec. Secretary, Dr. J. Howes.

CENSORS.

Dr. Arnold, Dr. J. F. Sandford, Dr. Hughes, Dr. Huey, Dr. J. F. Henry, Dr. Craig, Dr. Siveter.

Librarian, Dr. J. H. Rauch.

On motion, the report was received and adopted.

The reports of committees being now in order, the following were presented.

*Anatomy*—Reports by Drs. Hughes and J. F. Henry. Read and accepted.

*Surgery*—Dr. Sanford not being able to be present, transmitted to the Society a report of three cases of Lithotomy and one of fracture, which were read and accepted.

Society adjourned to meet at 7½ P. M.

7½ P. M. Society met according to adjournment.

On motion of Dr. Hughes,

Resolved, That when this Society adjourns, it adjourn to meet at Keokuk, on the 2d Wednesday of June, 1855, at 10 o'clock, A. M.

Dr. Henry here arose and announced the death of Drs. E. D. Ransom and J. W. Brookbank. Dr. M'Gugin reported the letter of condolence to the family and friends of Prof. Richards, dec'd.

Pending a motion of Dr. Rankin that this Society resolve itself into a committee of the whole, to devise means for the suppression of quackery and the promotion of good feeling among the members of the profession, Dr. Robertson was called to the Chair, and Dr. Reeder after a short dissertation upon the state of the profession, introduced the following,

Resolved, That this Society urge upon the physicians throughout the State, the importance of county organizations, the object of which shall be the promotion of medical science, and the encouragement of more thorough medical literature, by discountenancing and discouraging young men from entering the profession without sufficient pupilage. Adopted.

The committee appointed to nominate delegates, nominated the following gentlemen as delegates to the American Medical Association: Drs. Wainwright, M'Gugin, Chambers, J. F. Sanford, J. F. Henry and Reeder.

On motion, the committee on nominations reported the following committees for the ensuing year:

*Anatomy*—Drs. Arnold, Craig, and Rankin.

*Physiology*—Drs. Chambers, B. W. Thompson, and Wainwright.

*Therapeutics*—Drs. Witherwax, Cleaver, and Rauch.

*Pathology and Practice*—Drs. Robertson, M'Gugin, and Wear.

*Surgery*—Drs. Hughes, Reeder, and Sanford.

*Obstetrics*—Drs. J. F. Henry, Cleves, and Huey.

*Cholera*—Drs. M'Gugin, Waters, and Howes.

*Quackery*—Reeder, G. R. Henry, and Siveter.

On motion of G. R. Henry, an order was drawn on the treasury for a sufficient amount to pay the indebtedness of the Society, for the publication of the proceedings of the two preceding years.

On motion of Dr. Hughes, that a committeee be appointed to visit and examine into the method of teaching at the medical Schools in this State, the following committee was appointed: Drs. Reeder, Wainwright, Hershey, Skinner, Craig, and G. R. Henry.

On motion, the Society adjourned to meet at Keokuk, on the second Wednesday of June next.

J. HOWES, Rec. Sec'y.

---

**MEETING OF THE SOCIETY AT KEOKUK, THURSDAY JUNE 14TH, 1855.**

The sixth Annual Convention of the State Medical Society convened at the Medical Hall at this place pursuant to adjournment. The President and Secretary both being absent, Dr. Siveter was called to the Chair, and Dr. Craig of Van Buren Co. chosen Secretary temporarily.

The names of the following gentlemen were then presented for membership and were duly elected as members of the Society, viz: Drs S H Sawyers, of Appanoose Co; John R Allen, M F Collins, J J Page, R H Wyman, and J S Martin, of Keokuk; H J Scoles, of Charleston; Joseph H Smith, Farmington; Edward W. Laws, Oceola, Clark Co; Corydon Allen, Bentonsport; Hinsey of Delonega, and W Bird of Mount Pleasant, Henry County.

The Secretary being absent with the proceedings of last year, no reference could be had in the present proceedings to the past. The members therefore were called upon for miscellaneous reports of cases of practice.

Dr. Siveter reported a case of dislocated shoulder of some three months standing, which elicited some discussion.

Dr J F Sanford reported several cases of surgery of much interest to the profession; after which the Society adjourned to Friday morning at 9 o'clock.

FRIDAY MORNING.

The Society called to order by the President in the Chair, when on motion of Dr Arnold, a committee of three were appointed to nominate permanent officers of the Society for the ensuing year, who after retiring for a short time, reported the following names for officers, who were duly elected, viz:

President, Dr Thomas Siveter, Salem,; 1st Wice President, Dr Rauch, Burlington; 2d Vice President, Dr Craig, Keosauqua; Rec Secretary, Dr W Bird, Mount Pleasant; Cor Secretary, Dr D Atkinson, Dover, Lee Co; Treasurer, Dr J J Page, Keokuk.

CENSORS.

Dr. R. H. Wyman, Keokuk; Dr. T. Shriner, Salem; Dr. J. O. Skinner, Charleston, Lee county; Dr. J. G. Henny, Burlington; Dr. Hinsey, Dalonega.

Dr. Atkinson of Dover, Lee county; Dr. Kirkpatrick of Ottumwa, Dr. J. Haynes of Keokuk, and Dr. J. C. Wells of Indianola, Warren county, were then admitted to membership in the Society.

The following communication was then received from the members of the Faculty at Ft. Des Moines, which was read and ordered to be published with the proceedings of the Society:

FORT DES MOINES, IOWA,

June 5, A. D. 1855.

PROF. D. L. M'GUGIN,

Cor. Sec'y, Iowa Medical Society:

We, the undersigned Physicians and Surgeons at Fort Des Moines, in reply to your esteemed favor of the 29th May, soliciting our presence and participation in the proceedings of the Iowa State Medical Society at Keokuk, on the 14th inst., respectfully reply, that our professional avocations and private business, will prevent our attendance at the time specified, though we are decidedly in favor of the State organization, and of the Medical Department of the University of Iowa at Keokuk, and would be much pleased to participate in the proceedings of the State Medical Society, and further the interests of the State Medical College, whenever opportunity presents, We likewise approve of the Medical Journal, under the patronage and supervision of the Faculty of the Medical College of the University, as well calculated to promote the interests of the medical profession of Iowa.

JOHN C. BENNETT, M. D.

D. V. COLE, M. D.

THOMAS K. BROOKS, M. D.

WM. P. DAVIS, M. D.

J. W. MORRIS, M. D.

ALEX. SHAW, M. D.

S. V. CAMPBELL, M. D.

H. C. GRINNELL, M. D.

On motion, it was

*Resolved*, That the next annual meeting of the Society be held at Ottumwa, on the second Wednesday of June, 1856, at 2 o'clock P. M.

Dr. Allen of Keokuk, then offered the following series of Resolutions, which, after some discussion, were unanimously adopted;

WHEREAS, Being, as we are, fully aware of the great good derivable from a proper organization of the medical profession into State and subordinate associations, both in a scientific and social aspect, therefore

*Resolved*, That we, as members of the Iowa State Medical Society, and as individual members of the profession, will use every means in our power to increase the members and advance the objects of said societies.

*Resolved*, That we fully recognise the importance, and earnestly recommend to our members, the constant habit of recording their experience in practical medicine, and most urgently request that the results of such experience be made known to the medical profession generally.

*Resolved*, That as a means of publishing for mutual advantage what may be learned in private practice, we know of no more efficient, than their open statement and free discussion at the meetings of our various societies.

*Resolved*, That as a mode of giving permanency to these important developements, we recognise in the Iowa Medical Journal, a convenient medium in our midst, and which we feel it as a matter of State pride and professional interest to sustain, as the exponent of our medical literature.

A committee of three was then appointed to nominate Delegates to the National Medical Convention to be held at Detroit next year, who reported the following names:

Dr. Fountain, of Davenport; Dr. J. C. Bennett, of Fort Des Moines; Dr. Atkinson, of Lee; Dr. G. R. Henny, of Des Moines; Dr. W. Bird, of Henry county; Dr. Haynes, of Keokuk; who were duly elected.

Dr. Skinner, from the committee appointed at the last annual meeting to visit the Medical College during the last session, made the following report:

The undersigned of the committee appointed at the last annual meeting of the Iowa State Medical Society, begs leave to Report, That I visited the Medical Department of the Iowa Universisy, and heard the Professors several times, and from the ability with which each sustained himself in his respective department, it gives me pleasure to recommend them as men eminently competent to their several stations they so honorably fill.

The College is well supplied with the necessary appliances for teaching, to which, I was well assured from what I saw, the Faculty are adding liberally from time to time.

J. A. SKINNER, M. D., Committee.

Dr. Arnold, from the committee on anatomy, made an informal report, which, on motion, was received and the committee continued, with instructions to make a more full report at the next annual meeting.

Dr. Hughes, from the committee on surgery, being prevented from meeting with the Society by sickness, a portion of his report was read by Dr. Arnold, and the committee continued.

Dr. M'Gugin read a report of cases of tubercular disease, with treatment by the use of Cod Liver Oil, and inhalation of Iodine vapor.

On motion, Dr. Allen, of Keokuk, was appointed to deliver a public address at the next annual meeting.

On motion, the Secretary was ordered to procure two hundred copies of the Constitution and Bye-Laws of the Society printed for its future use. And ordered further, that the Secretary be requested to furnish a copy of the unpublished proceedings of the Society for the past and present meetings, to the editors of the Medical Journal at Keokuk, for publication ; said editors to furnish a copy of the Journal to each member of the Society, and draw on the Treasurer for the necessary cost of such publication.

On motion, Society adjourned to 2 o'clock, P. M.

FRIDAY AFTERNOON, 2 O'clock.

Society met.

Dr. M'Gugin moved that a committee of three be appointed to examine and endorse on behalf of the Society, the report of Dr. Arnold on the Medical Topography of the State for the use of the American Medical Association. Motion prevailed, and Drs. M'Gugin, Allen and Craig, appointed said committee.

The subject of memorializing the Legislature on the subject of legalizing the medical practice in the State, then came up, the discussion of which consumed most of the afternoon, when, on motion, the subject was referred to a special committee of three, who were directed to prepare a memorial to the Legislature, and present it to the next meeting of the Association for its concurrence.

Drs. D. L. M'Gugin, J. R. Allen, and W. Bird were appointed said committee.

The Society then adjourned *sine die.*

THOMAS SWETER,

President.

W. BIRD,

Secretary.

